# The structure of tropical bat–plant interaction networks during an extreme El Niño‐Southern Oscillation event

**DOI:** 10.1111/mec.16363

**Published:** 2022-02-15

**Authors:** Hernani F. M. Oliveira, Rafael Barros Pereira Pinheiro, Isabela Galarda Varassin, Bernal Rodríguez‐Herrera, Maria Kuzmina, Stephen J. Rossiter, Elizabeth L. Clare

**Affiliations:** ^1^ 4617 School of Biological and Chemical Sciences Queen Mary University of London London UK; ^2^ Departamento de Biologia Animal Universidade Estadual de Campinas Campinas Brazil; ^3^ Departamento de Botânica Universidade Federal do Paraná Curitiba Brazil; ^4^ Escuela de Biología, Centro de Investigaciones en Biodiversidad y Ecología Tropical Universidad de Costa Rica San Jose Costa Rica; ^5^ Centre for Biodiversity Genomics Biodiversity Institute of Ontario University of Guelph Guelph Canada; ^6^ Department of Biology York University Toronto ON Canada

**Keywords:** bat–plant interactions, climatic change, DNA barcoding, interaction networks, rainfall

## Abstract

Interaction network structure reflects the ecological mechanisms acting within biological communities, which are affected by environmental conditions. In tropical forests, higher precipitation usually increases fruit production, which may lead frugivores to increase specialization, resulting in more modular and less nested animal–plant networks. In these ecosystems, El Niño is a major driver of precipitation, but we still lack knowledge of how species interactions change under this influence. To understand bat–plant network structure during an extreme El Niño‐Southern Oscillation event, we determined the links between plantivorous bat species and the plants they consume by DNA barcoding seeds and pulp in bat faeces. These interactions were recorded in the dry forest and rainforest of Costa Rica, during the dry and the wet seasons of an extreme El Niño year. From these we constructed seasonal and whole‐year bat–plant networks and analysed their structures and dissimilarities. In general, networks had low nestedness, had high modularity, and were dominated by one large compartment which included most species and interactions. Contrary to our expectations, networks were less nested and more modular in drier conditions, both in the comparison between forest types and between seasons. We suggest that increased competition, when resources are scarce during drier seasons and habitats, lead to higher resource partitioning among bats and thus higher modularity. Moreover, we have found similar network structures between dry and rainforests during El Niño and non‐El Niño years. Finally, most interaction dissimilarity among networks occurred due to interaction rewiring among species, potentially driven by seasonal changes in resource availability.

## 1 INTRODUCTION

El Niño is one of the main drivers of precipitation fluctuations globally and is responsible for increasing differences in rainfall in the dry vs. wet season (seasonality) in the tropics (Holmgren et al., 2001). Such responses, however, differ widely among regions (Holmgren et al., 2001); for example, in parts of Central America, El Niño causes floods in the rainforests of the Caribbean coast, but droughts in Pacific dry forests (Waylen et al., 1998). These contrasting effects are critically important as rainfall (amount of annual rainfall and its seasonal distribution) is a major factor influencing plant phenology and primary productivity (Borchert, 1998). The frequency of strong El Niño/Southern Oscillation (ENSO) events is expected to increase with climate change (Cai et al., 2014). Indeed, the cycle of 2015–2016 is one of the strongest on record (Jacox et al., 2016). Although ecological responses to extreme weather events can be complex (Butt et al., 2015), changes in weather due to El Niño, including both droughts and floods, have been directly linked to fluctuations in fruit production (Wright et al., 1999), with cascading effects for wild animal and plant populations (Butt et al., 2015; Wright et al., 1999). Such impacts of El Niño might be especially important in the humid tropics, where nectarivorous and frugivorous vertebrates perform much of the pollination and seed dispersal and rely on plants for food, but these consequences have been little studied and remain poorly understood (Fredriksson & Wich, 2006; Wolfe et al., 2015; Wright et al., 1999).

The construction of ecological networks is a useful analytical approach for studying interactions among taxa across ecosystems (Ings et al., 2009). Most networks composed of plant and frugivorous or nectarivorous animals share similar properties. In particular, mutualistic networks are usually highly connected, with a single or a large compartment (Guimarães, 2020), and show nestedness, a tendency of interactions involving specialist taxa to represent a subset of those involving generalists (Bascompte et al., 2003). Moreover, they are also often modular, with multiple weakly linked clusters of densely connected taxa (Donatti et al., 2011; Olesen et al., 2007). Despite nestedness and modularity being negatively correlated in real‐world networks (Thébault & Fontaine, 2010), they may coexist within a hierarchical compound topology: a modular network with internally nested modules (Lewinsohn et al., 2006; Olesen et al., 2007). For example, a compound topology has been recently confirmed in a mutualistic animal–plant network in the tropics (Mello et al., 2019).

Topological patterns reflect underlying ecological and evolutionary processes driving species interactions in natural communities (Maynard et al., 2018; Pinheiro et al., 2019). Nestedness usually results from factors that unbalance the number of interactions made by each species (Guimarães, 2020), such as differences in abundances (Vázquez et al., 2007), and tends to be higher in networks dominated by generalist species (Pinheiro et al., 2019) or formed by weaker interactions (Fontaine et al., 2011). Modularity, on the other hand, requires discrete preferences among species. Module limits often reflect biological constraints that derive from functional or phylogenetic specialization (Donatti et al., 2011; Mello et al., 2019). Additionally, beyond revealing underlying mechanisms, topologies may relate to network stability and dynamics, affecting, for instance, robustness to the loss of species (Memmott et al., 2004), the influence of indirect effects (Guimarães et al., 2017) and the intensity of interspecific competition (Bastolla et al., 2009).

A major challenge in constructing networks is characterizing the links between species (Clare, 2014). Many vertebrate frugivores feed on fruit pulp, egesting no identifiable material (e.g., seeds) for morphological examination. For these reasons, DNA barcoding, which can be applied to traces of DNA, has proven to be a powerful means of inferring ecological interactions (Clare, 2014; Evans et al., 2016). Such molecular approaches have resolved previously unknown links in already well‐studied food webs, revealing that metrics such as connectance and nestedness may differ by orders of magnitude from earlier estimates derived from traditional approaches (Wirta et al., 2014). On the other hand, most studies using molecular tools to analyse animal diets have focused on predation (Jedlicka et al., 2013; Kruger et al., 2014) with fewer studies using DNA barcoding to understand mammal–plant interactions, though this is rapidly changing (Clare et al., 2019; Kartzinel et al., 2015).

In the neotropics, phyllostomid bats are widespread and critically important pollinators and seed dispersers, and, together with frugivorous birds, account for over 80% of seed dispersal activity (Galindo‐González et al., 2000). Previous work suggests bat–plant networks in the neotropics are highly connected, nested and robust to plant extinctions (Mello et al., 2011). Such network structures imply considerable behavioural flexibility that might confer resilience to changes in the environment, yet it is not known how extreme climatic events may affect the structure of these networks.

During the El Niño event of 2015, annual rainfall levels in the wet forest of Costa Rica exceeded those of the previous 47 years, whereas the opposite trend was observed in the coastal dry forest, where annual rainfall levels were lower than those of the previous 31 years. Moreover, most of the temporal differences in rainfall were related to changes in the rainy season for both forest types. Thus, the networks analysed here depict interactions in both types of forest during extreme climatic conditions associated with El Niño (Jacox et al., 2016; Seneviratne et al., 2012). As the frequency of extreme climatic events is expected to increase with climate change (Cai et al., 2014), knowledge of how networks are structured during these events becomes of utmost importance. Using a molecular approach, we describe the network of interactions between neotropical bats and plants in different seasons of an extreme ENSO year. While most of these are expected to be mutualistic interactions, some may represent herbivory, and thus we refer to these as bat–plant interactions rather than mutualism (see Discussion). Earlier work indicates network structure is strongly influenced by precipitation (Trøjelsgaard & Olesen, 2013). In general, increased seasonality (stronger changes in rainfall between the rainy and dry season) in comparison to non‐El Niño years, and higher annual rainfall are correlated with more modular networks (Dalsgaard et al., 2013; Schleuning et al., 2014; Trøjelsgaard & Olesen, 2013), and lower rainfall with greater nestedness (Rico‐Gray et al., 2012), probably as a result of changes in resource availability. As resources increase during higher rainfall, species become more specialized, which tend to increase network modularity, and decrease the interaction redundancy, with a consequent reduction in nestedness (Dalsgaard et al., 2013; Rico‐Gray et al., 2012; Trøjelsgaard & Olesen, 2013). A similar mechanism occurs in more seasonal environments, where species tend to become specialized in different resources following their availability across the year, leading to higher network modularity (Schleuning et al., 2014).

Bats number over 1,440 species worldwide, of which ~20% feed on nectar or fruit (Kunz et al., 2011; Mammal Diversity Database, 2021). Here we focus on interactions between plantivorous bats and plants in Costa Rica as a model to understand the structure of these networks during an extreme ENSO event in different habitats and seasons. To determine how opposite extremes in rainfall (unusually wet and dry conditions) induced by El Niño influence interactions among plants and plantivorous bats, we analysed and compared networks of interactions across the wet and dry seasons in both wet forest and dry forest in Costa Rica. Additionally, we contrasted this with a network of interactions during the wet season of the dry forest during a non‐El Niño year when annual precipitation was close to the average across years. We hypothesized that networks will show higher modularity and lower nestedness in the wet forest than in the dry forest. Similarly, within each forest type, we predicted that wet seasons would have higher modularity and lower nestedness than dry seasons. Additionally, because interactions are known to change according to ecological gradients (Poisot et al., 2012), we assessed the dissimilarity of the networks between seasons and between forests, accounting for both the species and the interaction dissimilarity.

## 2 MATERIALS AND METHODS

### 2.1 Study sites

**FIGURE 1:**
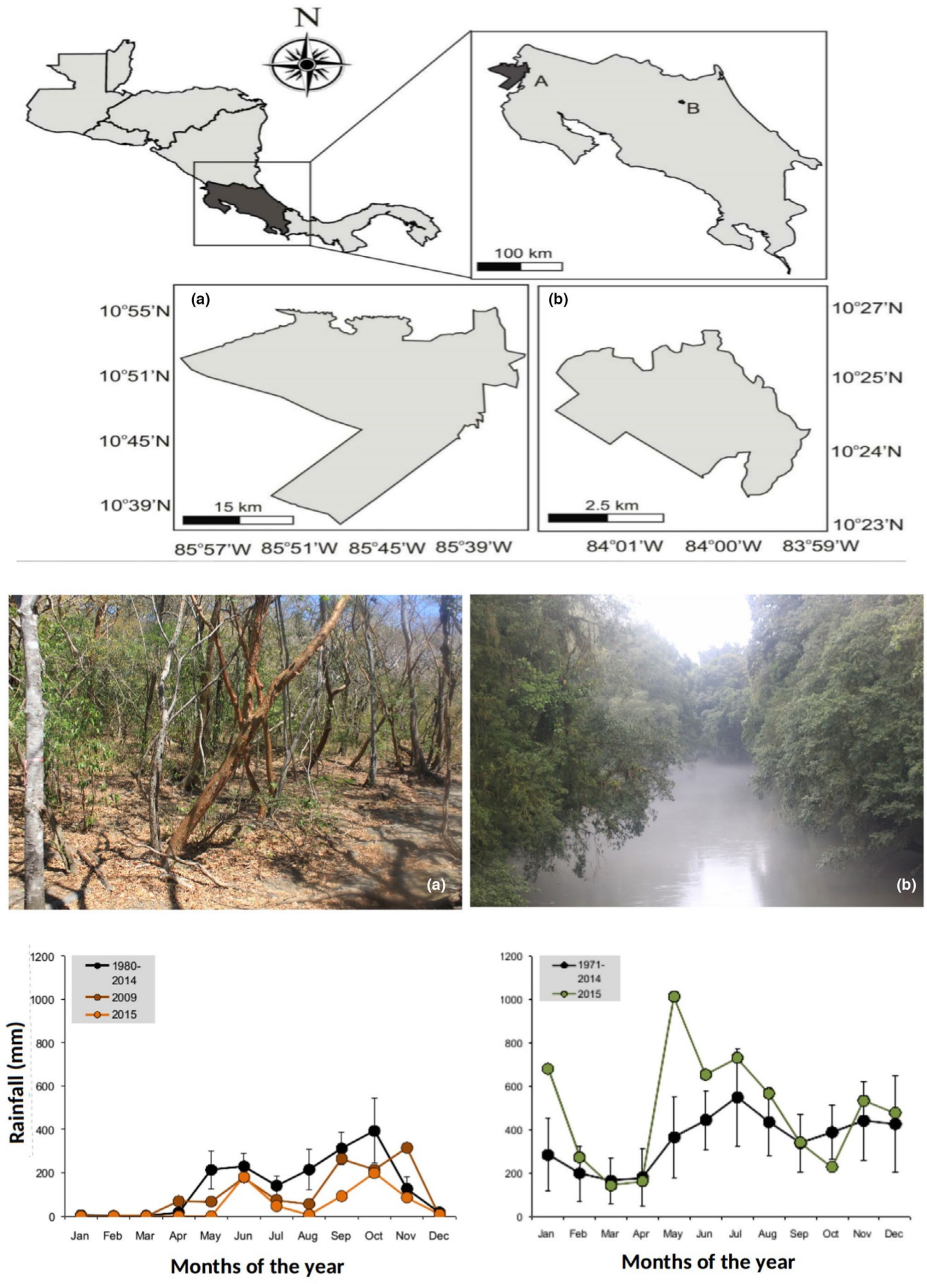
Map of Central America with Costa Rica and the field sites of the present study highlighted together with their monthly rainfalls. (a) Dry forest—Área de Conservación Guanacaste; (b) rainforest—La Selva Biological Station. Bars represent the standard deviation for the precipitation during each month

Fieldwork was conducted at two forest sites in Costa Rica that show contrasting seasonality and precipitation: the Atlantic rainforest of La Selva Biological Station (10°25′19″N, 84°00′54″W) and the Pacific dry forest at Sector Santa Rosa of Área de Conservación Guanacaste (ACG) (10°48′53″N, 85°36′54″W) (Figure 1). La Selva Biological Station covers 1,611 ha of lowland wet tropical forest between 35 and 137 m on the Caribbean slope of the Cordillera Central mountain range. It has a mean annual temperature of 25°C with a mean annual precipitation of 3,962 mm (Sigel et al., 2006). La Selva Biological Station has a mild dry season that ranges from January to April and a wet season that goes from May until December (Sanford et al., 1994). Sector Santa Rosa (of ACG) covers >38,000 ha of tropical dry forest ranging from 0 to 300 m, and is part of Área de Conservación Guanacaste (Asensio et al., 2015). Sector Santa Rosa (of ACG) has a mean annual temperature of 25°C with a mean annual precipitation of 1,575 mm. The dry season ranges from December to April, while the wet season extends from May to November, during which 85%–97% of the precipitation falls (Castro et al., 2018) (Figure 1). Annual precipitation is higher at La Selva Biological Station (range 2,809–6,164 mm) than at ACG (range 880–3,030 mm, 6‐month dry season) (Gillespie et al., 2000).

### 2.2 Bat sampling

We captured bats using four to six mist‐nets (6–12 m) opened along trails and near watercourses in the study area from 6 to 10 p.m. In addition, a canopy net and harp trap were used in 2009 but these had low capture rates and so were not used in 2015. Sampling took place in the wet season during May–July (Santa Rosa of ACG) (2009), and in the wet season during July–August (Sector Santa Rosa of ACG) and September–October (La Selva) (2015), and in the dry season during January–February (Sector Santa Rosa of ACG) and March–April (La Selva) (2015). Sampling and bat identification during the non‐El Niño year was conducted as described in Clare et al. (2019). Sampling effort using mist‐nets was equal to ~2250 m^2^ h within each season during the El Niño year, and approximately the same during the non‐El Niño year. We collected wing punches for another study and these also served to avoid recaptures in order to maintain the independence of the data in our analysis. We measured the forearm length with callipers (0.1‐mm precision) and identified species following Reid (1997), Timm and LaVal (1998), and LaVal and Rodriguez‐Herrera (2002). Bats were held in cloth bags for a maximum of 2 h for the collection of faecal samples. All samples were frozen after collection (−20°C).

### 2.3 DNA extraction, PCR and sequencing

For this study, we focused on nectar‐ and fruit‐eating species, which produced faecal samples consisting of either seeds or digested fruit pulp. For DNA extraction, PCR (polymerase chain reaction) and sequencing of the samples we followed standard protocols for plants and all work was conducted by the Canadian Centre for DNA barcoding (CCDB) following these procedures (Ivanova et al., 2011). In brief, dried plant material from faeces (fruit pulp or seed) was placed in a sterile strip‐tube with pre‐aliquoted sterile stainless steel beads and the tissue was ground using a Tissue Lyser (Qiagen). The ground material was incubated with 2× CTAB buffer at 65°C for 1 h and DNA extraction was performed using a semi‐automated glass fibre filtration method (Fazekas et al., 2012; Ivanova et al., 2008). Following established methods, we amplified a 552‐bp fragment of the 5′ end of the large subunit of RuBisCO (*rbcL*) and the ~350‐bp second nuclear encoded internal transcribed spacer (ITS2) flanked by the partial 5.6S and 26S ribosomal genes. Sanger sequencing was performed using a ABI 3730xl capillary sequencer (Fazekas et al., 2012; Ivanova et al., 2005; Ivanova & Grainger, 2006; Kuzmina & Ivanova, 2011a, 2011b). Although plant DNA barcoding yields lower species resolution compared to fungi and animals (Hollingsworth et al., 2011), generally it provides robust results for identification of vascular plants at the genus level (Braukmann et al., 2017; Kress et al., 2009; Parmentier et al., 2013). For the samples from the non‐El Niño year, plant specimens collected on the sites were identified using *rbcL* and *matK* and the supplementary noncoding plastid region *trnH*‐*psbA* (see Clare et al., 2019 for full methods).

### 2.4 Identification of plant DNA sequences from bat faecal samples

We initially filtered all sequences for quality and excluded low‐quality sequences where the PHRED score was <30 as indexed on the Barcode of Life Data Management System (BOLD) (Ratnasingham & Hebert, 2007). At least one genetic marker was recovered for each faecal sample, and thus no samples were excluded at this stage. We compared the obtained *rbcL* and ITS2 sequences with the reference libraries of GenBank and BOLD using the blast algorithm with default search parameters (Altschul et al., 1990) in GenBank and the combined blast and Hidden Markov Model methods implemented by the BOLD server (Ratnasingham & Hebert, 2007). For each reference database (BOLD, GenBank), we assigned query sequences to taxa based on the highest percentage similarity, and considered a threshold of ≥97% to be a reliable assignment (Lamb et al., 2016). When there was an agreement between species‐level matches for both markers (*rbcL* and ITS2) in both databases, with at least one match >97%, we assigned a species name. In cases where the query matched with equal similarity to multiple taxa in the same genus, we assigned the taxon to the level of genus only, and similarly we used the same approach to assign query sequences to the level of the family. Where *rbcL* and ITS2 sequences matched different species from different genera, both at >97%, we concluded that two taxa were present in the sample and assigned them to both genera. Query sequences that did not show significant similarity to a reference were excluded from the analysis, with only one faecal sample removed due to this procedure.

To corroborate species assignments, for each candidate genus match, we reconstructed a gene phylogeny in which we included our query sequences together with all available reference sequences from species of the same genus present in BOLD that are also known to occur in Costa Rica. Sequences from *rbcL* and ITS2 of each plant genus were aligned with clustalw (Larkin et al., 2007) in bioedit version 7.2.5 (Hall, 1999). For each alignment we ran a model selection test to check which would be the best method to build the phylogenetic tree based on the lowest Bayesian information criterion (BIC) value. We ran model selection and built the phylogenetic trees using mega 6.06 (Tamura et al., 2013). These phylogenies (not shown) recovered paraphyletic groupings for some species, perhaps through a lack of reference material, and therefore such species assignments were considered unreliable. To address this impact of taxonomic resolution, we reduced all data mostly to genus‐level designations and repeated our analyses for both species and genera data to check for consistency of results (see Supporting Information).

The identification of plant DNA sequences from bat faecal samples during the non‐El Niño year relied on GenBank and BOLD, with the exception of the *trn*H‐*psb*A region which was not searchable within BOLD (see Clare et al., 2019 for more details); for our purposes we used the assignments as given in Clare et al. (2019).

### 2.5 Network matrices

We compiled the inferred interactions into weighted matrices where each cell value represented the number of observed interactions between each bat–plant taxon pair. We considered one realized interaction when the DNA of a plant taxon was detected in the faeces of one individual bat. We constructed matrices for (i) each forest site in which we pooled data from both seasons during the El Niño year (“La Selva” and “Santa Rosa”), and (ii) for each forest site in which we separated the data collected for dry and wet seasons during the non‐El Niño and El Niño years (“wet” vs. “dry” for each site). As species usually distribute their interactions unevenly among partners (Ings et al., 2009), weighted analysis might uncover preferences, and even modules, that are invisible to binary analysis. Thus, we additionally applied weighted metrics to characterize network topologies. All statistical analysis and network drawings were performed using r version 3.3.2 (R Development Core Team, 2017).

### 2.6 Descriptors of network structure

To determine network structure from each habitat during a whole El Niño year, and for each habitat during each season during the El Niño and non‐El Niño year, we assessed network structure by measuring five key metrics. First, we quantified nestedness, which measures the extent to which the interactions of one species are a subset of the interactions of another species when the matrix of interaction is organized by decreasing number of links (Dormann et al., 2009). We calculated nestedness using the WNODF index, which is a measure of nestedness that uses overlap and decreasing fill in the weighted matrix, and has been shown to outperform other methods for estimating nestedness in binary networks (Almeida‐Neto & Ulrich, 2011). Second, we quantified modularity, characterized as more interactions within a module than between modules (Dormann & Strauss, 2014), using the DIRTLPAwb + algorithm that is based on bipartite modularity optimization and a multistep greedy agglomerative algorithm (Beckett, 2016). DIRTLPAwb + maximizes the density of edge weights within modules and is designed for weighted bipartite networks (Beckett, 2016). Besides that, it returns the solution which finds the greatest modularity score after calculating modularity with different random initializations (Beckett, 2016). In a highly modular network, nestedness between species of different modules is always reduced, but nestedness may still prevail within modules (Mello et al., 2019). Thus, third, we separately calculated the nestedness between species that belong to the same module (WNODF_SM_) (Felix et al., 2021). Fourth, we calculated network connectance, which is the proportion of potential links between species that were observed. Fifth, we calculated the number of compartments, which are defined as isolated subsets of nodes interacting with each other that do not have any connections with another compartment in the network (Dormann et al., 2009). All metrics chosen have moderate (number of compartments, connectance, modularity) or no biases (WNODF) to sampling completeness and network size (Fründ et al., 2015). In order to determine the sampling completeness of our networks and the proportion of the total plant species richness present in bat diets that have been sampled, we used the Chao 1 index for the networks (Chao, 1987; Chiu, 2014), and individual‐based rarefaction curves for the estimation of each bat species diet (See Supporting Information). All metrics were calculated using the bipartite package for r (Dormann et al., 2008).

### 2.7 Null model analysis

Null models are useful theoretical benchmarks to test whether the observed network structure can be explained by more simple features, such as network size and sampling effort, without invoking other causal mechanisms (Guimarães, 2020). Here, we have used the approach proposed by Vázquez et al. (2007) and operationalized through the *vaznull* algorithm in the bipartite package (Dormann et al., 2008) in order to build randomized matrices. This algorithm conserves the observed matrix dimensions, connectance and the total frequency of interactions, in the randomized matrices. It also probabilistically conserves the marginal sums, as the chance of each species receiving interactions during randomization is based on the observed marginal frequencies. We choose the *vaznull* algorithm because its constraints are straightforward and biologically meaningful, prompting a more informative comparison. Note, however, that *vaznull* tends to conserve the skewness of marginal sums, which are a known cause of nestedness, and conserves connectance, a feature that correlates with modularity. The absence of deviation between the observed and null model must not be understood as the absence of structure in the networks, but that network structure of our null models, which was generated according to these constraints, was enough to replicate our observed network structure (Guimarães, 2020). To compare WNODF_SM_ values we built an additional null model that, beyond the features conserved by *vaznull*, also conserves the original modular structure (Felix et al., 2021). For each observed matrix and each null model, we generated 1,000 random matrices and calculated *z*‐scores to check if the observed network metric value was higher or lower than expected given the features preserved by the null model.

### 2.8 Network dissimilarity

To better understand the effects of habitat and season on the composition and interactions of the networks during the ENSO event, we calculated the dissimilarity of species composition and interaction matrices between habitats and between seasons within each habitat using the r package betalink (Poisot et al., 2012). We first calculated the dissimilarity in the species composition of communities in the networks (*β*_S_). We then calculated the dissimilarity of the interactions (*β*_WN_) based on the differences in the interactions observed between species shared by both networks (*β*_OS_) and on the dissimilarity of interactions due to species turnover (*β*_ST_), using the function betapart from the betalink package (Poisot et al., 2012).

## 3 RESULTS

**FIGURE 2:**
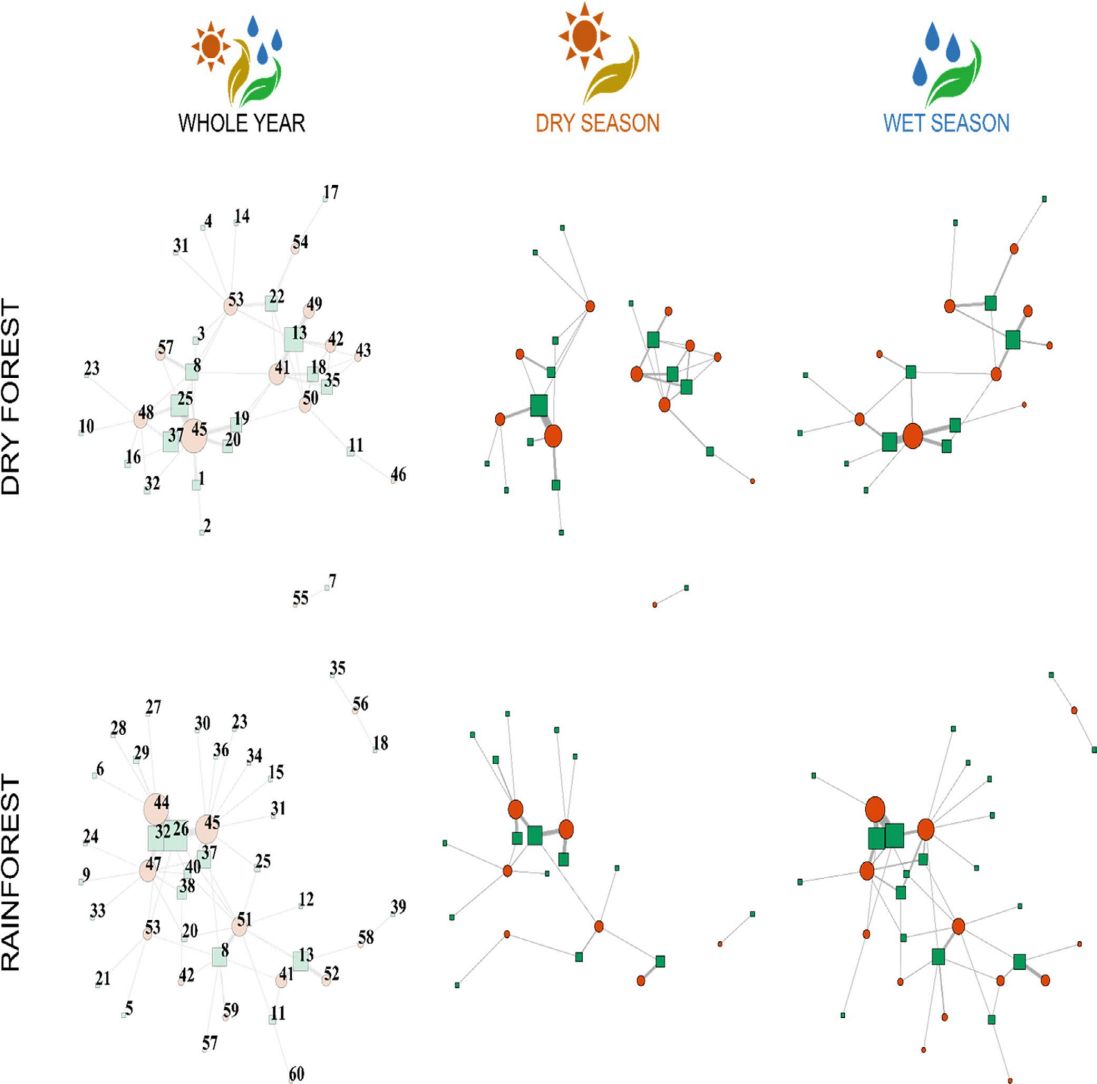
Force directed graphs of interaction networks between plantivorous bats (red circles) and the plant taxa (green squares) present in their diet in the dry forest of Sector Santa Rosa (of Área de Conservación Guanacaste) (Costa Rica) and in the rainforest of La Selva Biological Station (Costa Rica), during an extreme El Ninõ year (2015). Networks for the whole‐year and separated into the wet and dry seasons. Plant taxa were identified to lowest taxonomic level possible. 1, *Annona reticulata*; 2, *Bernardia nicaraguensis*; 3, *Bauhinia ungulata*; 4, *Bauhinia*; 5, Bromeliaceae; 6, *Columnea purpurata*; 7, *Casearia*; 8, *Cecropia*; 9, *Epipremmum*; 10, *Erythroxylum*; 11, *Ficus citrifolia*; 12, *Ficus dewolfii*; 13, *Ficus*; 14, *Helicteres*; 15, Juglandaceae; 16, *Karwinskia*; 17, *Muntingia calabura*; 18, *Manilkara chicle*; 19, *Maclura tinctoria*; 20, Moraceae; 21, *Ochroma pyramidale*; 22, *Piper amalago*; 23, *Piper auritum*; 24, *Piper glabrescens*; 25, *Piper marginatum*; 26, *Piper multiplinervium*; 27, *Piper peltatum*; 28, *Piper reticulatum*; 29, *Piper sancti‐felicis*; 30, *Philodendron*; 31, *Pinus*; 32, *Piper*; 33, *Pourouma*; 34, *Senna papillosa*; 35, Sapotaceae; 36, Saxifragaceae; 37, *Solanum*; 38, *Vismia macrophylla*; 39, *Vismia*; 40, Zingiberales; 41, *Artibeus jamaicensis*; 42, *Artibeus lituratus*; 43, *Dermanura tolteca*; 44, *Carollia castanea*; 45, *Carollia perspicillata*; 46, *Centurio senex*; 47, *Carollia sowelli*; 48, *Carollia subrufa*; 49, *Chiroderma villosum*; 50, *Dermanura phaeotis*; 51, *Dermanura watsoni*; 52, *Ectophylla alba*; 53, *Glossophaga* sp.; 54, *Lonphophylla robusta*; 55, *Micronycteris microtis*; 56, *Phyllostomus discolor*; 57, *Platyrrhinus helleri*; 58, *Uroderma convexum*; 59, *Vampyriscus nymphaea*; 60, *Vampyressa thyone*

In 130 sampling nights during the El Niño year, we captured 1,041 bats from 42 species, and collected guano samples from 435 individuals of 20 plantivorous species. Analyses of faecal material from these 20 bat species recovered a total of 40 plant taxa, representing a total of 257 observed interactions. Of these 40 taxa, 20 plant taxa were resolved to species, 14 to genus, five to family and one to order (though see also the Supporting Information for analysis of genera) (Figure 2). From the non‐El Niño year data set (Clare et al., 2019), we captured a total of 801 bats from 26 species over 6 weeks of sampling, and collected guano samples from 112 plantivorous individuals of 12 species. Analysis of faecal material from these 12 bat species recovered a total of 20 plant taxa, representing a total of 117 observed interactions. Of these 20 taxa, 13 plant taxa were resolved to species, and seven to genus. Values of sampling completeness of networks ranged from 78.85% (dry forest—non‐El Niño wet season) to 95.65% (rainforest—dry season) (see Supporting Information).

### 3.1 Network structure in dry forest and rainforest during an El Niño year

**FIGURE 3:**
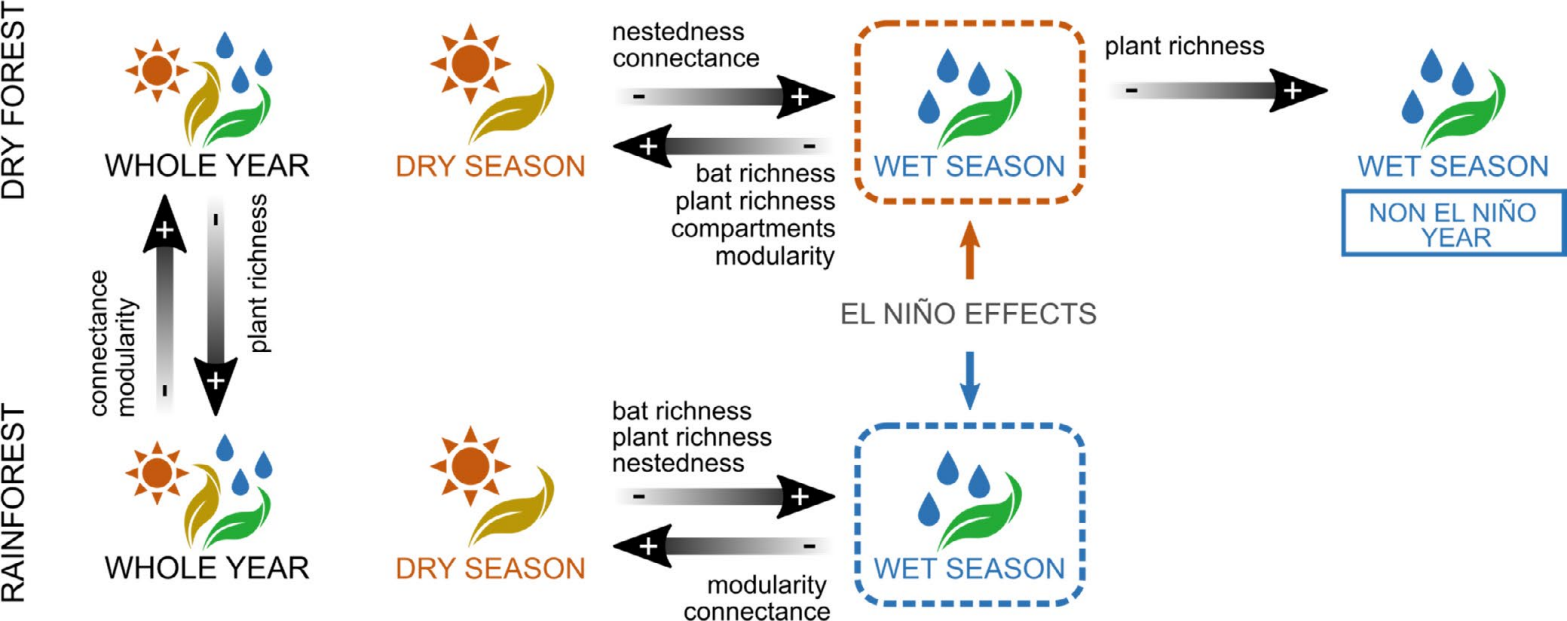
Main differences in the structure of bat–plant networks described in our study. Comparisons are between networks in different forest types, dry forest and rainforest, and between seasons within each forest type, during an extreme El Ninõ year (2015). The El Niño had opposite effects for each forest type, namely increased drought in the dry forest and increased rainfall in the rainforest, which mostly occurred during the wet season in both cases. Additionally, we compared the mutualistic network in the dry forest during the wet season of 2015 to the corresponding network in a non‐El Niño year (2009)

Both the dry forest and the rainforest formed connected networks (*sensu* Guimarães, 2020) composed of a large and a very small compartment (formed by nodes 55 and 7 in the dry forest, and nodes 35, 56 and 18 in the rainforest) (Figure 2) with low overall nestedness and high modularity (Table 1). In both cases, observed nestedness was lower but modularity was higher than in the randomized matrices of the null model. Moreover, we found that the whole‐year dry forest network was more modular than the whole‐year rainforest network (Figures 2 and 3; Table 1). Additionally, the rainforest network presented a higher species richness (42 vs. 34), mainly because of plant richness (29 vs. 22), and lower connectance.

**TABLE 1:** Bat–plant network metrics of networks in the wet and dry season of the dry forest of Sector Santa Rosa (of Área de Conservación Guanacaste) and rainforest of La Selva Biological Station in Costa Rica during an extreme El Niño year (2015) and a dry forest wet season of a non‐El Niño year (2009)

Network metric	Dry forest				Rainforest		
	Whole‐year	Dry	Wet	Wet non‐El Niño (2009)	Whole‐year	Dry	Wet
Plant richness	22	16	12	20	29	16	20
Bat richness	12	11	9	10	13	7	13
Number of compartments	2	3	1	1	2	2	2
WNODF	0.14 (−2.1)	0.07 (−2.4)	0.17 (−0.7)	0.15 (−2.3)	0.14 (−2.1)	0.07 (−1.4)	0.15 (−1.1)
Modularity	0.53 (5.9)	0.56 (2.8)	0.52 (2.1)	0.51 (6.4)	0.46 (3.7)	0.55 (0.7)	0.48 (2.3)
WNODF_SM_	0.33 (−1.9)	0.23 (−1.4)	0.48 (0.3)	0.32 (−0.1)	0.37 (0.3)	0.28 (0.2)	0.37 (2.0)
Connectance	0.19	0.18	0.21	0.21	0.15	0.20	0.16

Note

For the topological metrics, *z*‐scores compared to the null model are given in parentheses.

### 3.2 Network structure in wet and dry seasons during an El Niño year

In general, seasonal networks formed connected networks, with a single large compartment including all or most species (Table 1). Networks conformed more to a modular than to a nested topology. Modularity values ranged from 0.46 to 0.56 and, except from the dry season of the rainforest, were higher than observed in the null model. Nestedness values were generally low, ranging from 0.07 to 0.17. Nestedness between species in the same modules was also not high in most of the networks (0.23–0.48) and was closer to values in the null model.

In the dry forest during the El Niño year, the network of interactions was richer during the dry season than during the wet season, including both more bats and more plants. The most relevant difference between these networks was that during the dry season the network was broken in three compartments, two large and one small (Figure 2). Furthermore, the dry season network had higher modularity and lower nestedness than the wet season network (Figure 3; Table 1).

In the rainforest, the network of interactions was richer in plant and bat species during the wet season. However, the dry season had higher modularity and connectance, but lower nestedness than the wet season network (Figure 3; Table 1).

### 3.3 Network structure in the wet season during a non‐El Niño year

The topology of the network in the dry forest during the wet season of 2009, a year without an El Niño event, was very similar to the wet season of 2015 in the same forest type, with most metrics showing very small variations (Table 1). The only relevant differences were higher plant richness (20 vs. 12) in the network of 2009 and a higher WNODF_SM_ (0.48 vs. 0.32) in the network of 2015 (Figure 3).

### 3.4 Network dissimilarity across forests and seasons

**FIGURE 4:**
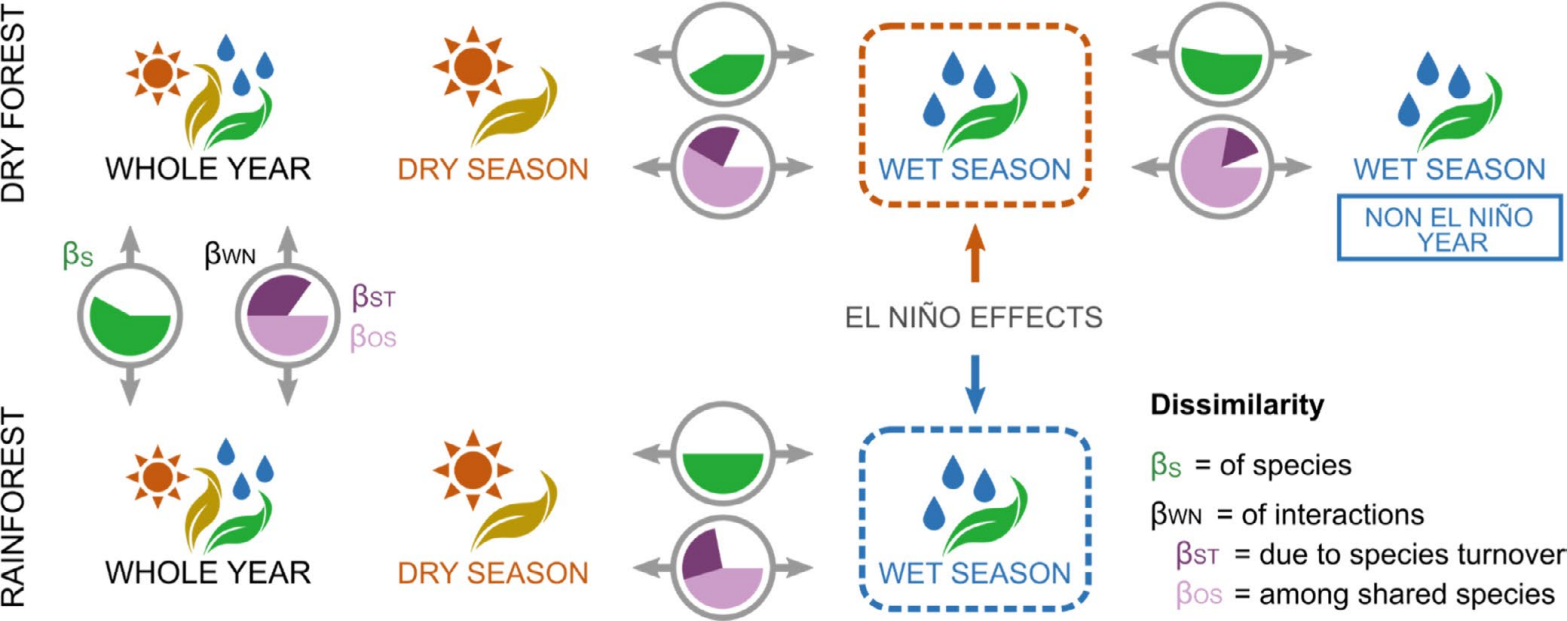
The dissimilarities of species (*β*_S_) and of interactions (*β*_WN_) between bat–plant networks described in our study. Here, the dissimilarity of interactions between networks is divided into two additive components: the dissimilarity due to species turnover (*β*_ST_) and the dissimilarity due to the rewiring of interactions among shared species (*β*_OS_). Comparisons are between networks in different forest types, dry forest and rainforest, and between seasons within each forest type, during an extreme El Ninõ year (2015). The El Niño had opposite effects for each forest type, namely increased drought in the dry forest and increased rainfall in the rainforest, which mostly occurred during the wet season in both cases. Additionally, we compare the mutualistic network in the dry forest during the wet season of 2015 to the corresponding network in a non‐El Niño year (2009)

The dissimilarity of species was higher between the different forests than between seasons within each forest (Figure 4; Table S1). All pairwise comparisons showed a high level of interaction dissimilarity (*β*_WN_ >0.7), with most of this dissimilarity arising from the rewiring of interactions between species shared by both networks and a smaller portion being due to species turnover (Figure 4). Moreover, the comparison between the El Niño and non‐El Niño year during the wet season in the dry forest revealed a higher dissimilarity of species, due to the dissimilarity of interactions among shared species, than any other comparison between seasons within the same forest type.

## 4 DISCUSSION

Our study demonstrates that DNA barcoding is a powerful tool to detect interactions, and it enabled us to reconstruct the networks of interactions between 20 plantivorous bat species and the many plant taxa they visit. Contrary to our initial prediction, networks from wetter habitats or seasons were less modular and showed more nestedness than networks from drier habitats or seasons. Moreover, most changes in interaction dissimilarity occurred due to interaction rewiring, potentially driven by changes in resource availability.

### 4.1 General features of the bat–plant networks

As expected, most networks were highly connected, with a unique large compartment including most or all species. The emergence of this configuration is associated with the presence of at least a few highly connected species (González et al., 2010), also termed supergeneralists (Jordano et al., 2003), which rely on resources that are common to the entire network (Guimarães, 2020). For all our networks, the bat species that best matches this concept is *Carollia perspicillata* (node 45, Figure 2) which consume fruits from more than 10 plant species in several different genera. Conversely, plants whose fruits are consumed by a large proportion of bat species, such as *Piper multiplinervium* in the rainforest (node 26, Figure 2), may also be considered supergeneralists. These highly connected species have a disproportionate impact on network structure (González et al., 2010), preventing fragmentation (Olesen et al., 2007), and allowing the propagation of trophic cascades and eco‐evolutionary feedback across species (Guimarães, 2020).

In general, our networks did not conform well to any topological pattern (Lewinsohn et al., 2006) and were more modular than nested (Olesen et al., 2007). Our networks do not fit to a unified archetype, such as the description of mutualistic networks as highly nested (Bascompte et al., 2003) but may show a wider range of structural variation and larger internal complexity.

Modularity usually results from biological constraints that promote preferences among species (Donatti et al., 2011). Because the studied networks are formed by interactions that do not require a strong physiological or morphological integration between partners (Fontaine et al., 2011), we expected the biological constraints to be weak and interactions to be more generalized and nested. However, the high modularity and low nestedness might be due to the high diversity of plants in the studied communities. In consumer–resource networks, when resources are too diverse and heterogeneous, consumers may face adaptive trade‐offs to exploit them (Krasnov et al., 2012; Pinheiro et al., 2019). In our case, trait mismatching between fruits (varying in size, shape or chemicals) and bats hampers some interactions (Dehling et al., 2016), and thus the network might include plants whose fruits are so different (for instance, in size, shape or chemicals) that each bat species cannot be efficient in finding and eating all of them, leading to the emergence of modules (Mello et al., 2019). Our results are similar to previous detections of modularity in bat–plant local networks in South America (Mello et al., 2011), but the higher modularity we found might be due to an increased detectability of interactions provided by DNA barcoding (Clare et al., 2014; Wirta et al., 2014). The detection of missing links and rare interactions, which was enabled by the use of DNA, can allow for greater differentiation between bat species’ diets.

### 4.2 Comparisons between networks

Because resource availability usually increases with higher rainfall in tropical forests (Rathcke & Lacey, 1985; van Schaik et al., 1993), we expected increased specialization and a higher species richness, decreasing connectance and promoting modularity over nestedness in the networks from wetter conditions (Dalsgaard et al., 2013; Rico‐Gray et al., 2012; Trøjelsgaard & Olesen, 2013). Indeed, in most cases, networks were richer and less connected in wetter than in drier habitats, with the main exception, the comparison between seasons in the dry forest (Figure 3), probably being affected by the precipitation shortage caused by the El Niño event during the wet season. However, in contradiction to our predictions, we found that networks of interactions between bats and plants in wetter habitats, both in dry forest and rainforest, and between seasons, are less modular and more nested than in drier habitats (Figure 3; Table 1). This might be due to lower resource availability in drier habitats leading to higher competition and resource partitioning among species (Chesson, 2000), such as found in other seasonal areas (Souza et al., 2018). In general, when fruits are abundant, bats have a broad diet, but when fruits become scarce, bat species reduce their consumption to a few plants. Additionally, as the phyllostomid bats present diverse feeding habits (Dumont et al., 2012), it is possible that other resources, such as nectar and insects, might help them to survive with a narrower fruit diet during fruit shortages (York & Billings, 2009).

Our findings that richer and less connected bat–plant networks were also more nested and less modular is unusual for the ecological literature, as the opposed relationship is most often found (Pinheiro et al., 2019; Thébault & Fontaine, 2010; Trøjelsgaard & Olesen, 2013). The simplest explanation for our observation is that when comparing networks, more species do not form new modules but instead were mostly added into already highly connected networks. This kind of preferential attachment probably occurs also in animal–plant mutualisms (Jordano et al., 2003; Olesen et al., 2008) and promotes the emergence of nestedness (Mello et al., 2019). The evidence for preferential attachment is observed in the comparison between seasons in the rainforest (Figure 2): the network during the wet season is basically a larger version of the network in the dry season, with the same central species making more connections (*Carollia castanea*, *Carollia perspicillata* and *Dermanura watsoni*). As a result, overall nestedness within modules increased (Mello et al., 2019) and supergeneralist species became even more generalist (Jordano et al., 2003).

The observed dissimilarity in species composition between the rainforest and dry forest is close to what has been observed for the species turnover between lowland dry forests and rainforests in Costa Rica, where 50%–100% of plants and animals were common to both forests (Janzen, 1986). Species dissimilarity (*β*_S_) was higher between forest types than between seasons within each forest (Figure 4; Table S1). Temporal changes in phyllostomid bat composition are probably related to the abundance of preferred food items and stratification (Mello, 2009). While species that forage in the canopy tend to be more specialized on tree species that produce high numbers of fruits for short periods of time, understorey bats feed mainly on plants that produce few fruits over many months of the year (Mello, 2009). Thus, the higher dissimilarity found between forests than between seasons within each forest might be linked to differences in plant composition between forest types rather than between seasons due to the constant production of fruits over the year by understorey plants. Moreover, seasonal differences in plant species composition of bat diets may be more related to resource use at the canopy level together with potential changes in fruit production driven by rainfall changes during the precipitation extremes of the El Niño.

We observed high interaction dissimilarity (*β*_WN_) between forests and between seasons in both forests (Figure 4). Similar observations have also been made for mutualistic networks over time (years) where the percentage of retained interactions was similarly low, ranging from 5% to 31% (Petanidou et al., 2008; Vázquez et al., 2009). In all comparisons, most of the dissimilarity came from the rewiring of interactions between species that are shared by both networks rather than from species turnover, which means that the same bat species eats fruits from different plants in different forests and seasons or the same plant species are dispersed by different bats across forests and seasons. While this observation is interesting, it should be noted that we have no replication in this observation and it should be considered preliminary until additional sites can be compared. This rewiring of interactions had clear structural consequences for the seasonal networks in the dry forest, connecting wet season compartments that were isolated during the dry season (Figure 2). It is likely that most of the dissimilarity between seasons within each forest type is caused by differences in fruit availability (Laurindo et al., 2017), which, at least for the dry forest, is known to show temporal variation (Kushwaha et al., 2011).

To understand exactly how El Niño affected the bat–plant networks is beyond the scope of our study, as we have little information on the networks for non‐El Niño years. When comparing the only available network in a non‐El Niño year, the dry forest in the wet season of 2009, to the respective network during the El Niño event, we found a higher richness in the non‐El Niño year and a high dissimilarity of interactions, although network structure remained similar. This robustness of network structure might be due to a high functional redundancy in the regional community, so that, under different circumstances, similar structural roles in the network are played by different species (Fonseca & Ganade, 2001). Nevertheless, we hope the networks we describe and explore here are useful assets for future studies on the effects of El Niño.

### 4.3 Limitations of the study

One caveat of our study, which is ubiquitous to studies of seed dispersal networks, is that we cannot be certain that every interaction recorded would lead to the successful dispersion of seeds. Even though most of the sequenced faecal samples were from seed material (>80%) and bats fly away from the mother tree to eat the fruits on feeding perches, which usually leads to seed dispersal, we cannot guarantee that all our samples resulted in seed movement away from the mother tree into suitable sites for germination. Among fruit bats, such as *Artibeus*, large seeds can be dispersed over long distances simply by being carried in the bat's mouth. For example, we have observed mango seeds sprouting deep in caves where they have been carried by large *Artibeus*. As a consequence, seeds need not be ingested to be dispersed in a mutualistic relationship with the bat (Laurindo et al., 2019; Marjakangas et al., 2018). We include pulp in our analysis. The advantage of DNA‐based approaches is that these traces can then be identified and included in the analysis, but we also run the risk of including frugivorous interactions alongside mutualisms. Even when this movement occurs, we cannot be certain of the final seed outcome, as it might be deposited in inadequate sites for germination or destroyed during gut‐passage; for example, the seeds described above germinated in the cave but could never develop. Thus, although most of our interactions are probably mutualistic, networks might also include interactions that do not result in benefit to the plants and thus we describe them as “bat–plant” interactions rather than mutualisms.

Another limitation of this study is it is hard to assess the influence of the choice of genetic markers that we have used for plant identification on the values of network metrics. Multiple genetic markers have been proposed in various combinations to identify different plant species (*matK*, *trnH*‐*psbA*, *rbcL*, ITS2), but are still not sufficient to discriminate closely related species in some taxonomic groups, especially those with recent and intense species radiation (Hollingsworth et al., 2011). For example, the fig tree (*Ficus*), one of the genera commonly consumed by bats, is extremely speciose and demonstrated poor resolution at the species level using *rbcL* and ITS (Ronsted et al., 2008). As such, species identity should be treated provisionally. However, our analysis of data limited mostly to genera (see Supporting Information) where identifications are much more robust suggests our observations are consistent even when taxonomic designations are constrained.

An additional limitation of our study is the lack of temporal and spatial replicates of the networks within each forest type. Even though our networks have been shown to be relatively well sampled in comparison to other network studies focused on seed dispersal, our lack of replication does not allow us to assess trends beyond our mentioned scales. Thus, we cannot anticipate how consistent our observations would be and how far they can be extrapolated to other regions and years.

Finally, sampling completeness is a common problem for network studies, with many studies relying on networks with sampling completeness estimates below 90%. Thus, while this should be treated with caution, our data are not unusual and we have tried to address this by focusing on network metrics that do not show a strong bias due to network size, which should minimize the impact of these issues.

## 5 CONCLUSIONS

Our study demonstrates that combining DNA barcoding with ecological networks is a powerful tool not only to uncover previously undetected interactions according to traditional identification methods, but also to analyse how ecological systems are structured during extreme climatic events. Moreover, we have shown that the structure of bat–plant interaction networks in the Neotropics tends to be similar when comparing dry and rain forests, and El Niño and non‐El Niño years. These findings are of paramount importance, since extreme climatic events, such as those that we have analysed in the current study, are likely to become more frequent and have strong consequences for changes in rainfall in many parts of the world. Thus, more studies are required to better understand how common the patterns we observed are across spatial and temporal gradients.

## CONFLICT OF INTEREST

The authors declare that there are no conflicts of interest in the publication of the current article.

## AUTHOR CONTRIBUTIONS

H.F.M.O., S.J.R. and E.L.C. designed the project; H.F.M.O., S.J.R., E.L.C. and M.K. identified plant DNA sequences; H.F.M.O. conducted fieldwork; H.F.M.O. and R.B.P.P. performed statistical analysis; H.F.M.O., R.B.P.P., I.G.V., B.H.R., M.K., S.J.R. and E.L.C. wrote the manuscript.

## Supporting information

Supplementary Material

## Data Availability

All sequence records are available on the BOLD website (www.boldsystems.org) in the public project HFACG. The R code for the analysis performed in the article are available on Github (https://github.com/pinheirobp/bat‐plant‐networks) (https://doi.org/10.5281/zenodo.5866787). The network matrices are available on DRYAD under the name “Molecular Food Webs of Bat‐Plant interactions during an extreme El Nino event” (https://doi.org/10.5061/dryad.0rxwdbs29) (Oliveira et al., 2020).
