# Supplementary material for: The structure of tropical bat–plant interaction networks during an extreme El Niño‐Southern Oscillation event
Source: Mol Ecol. 2022 Feb 15;31(6):1892–906. doi: 10.1111/mec.16363 (PMC9305221; doi:10.1111/mec.16363)
Supplement: Supplementary file 1 — Supplementary Material [file MEC-31-1892-s001.docx]

Supplemental Information for:

The structure of tropical bat-plant interaction networks during an extreme El Niño-Southern Oscillation event

Hernani F. M. Oliveira1*; Rafael Barros Pereira Pinheiro2; Isabela Galarda Varassin3; Bernal Rodríguez-Herrera4; Maria Kuzmina5; Stephen J. Rossiter1 & Elizabeth L. Clare1,6.

	Table of Contents:
Title page	Page 1
Figure S1	Page 2
Figure S2	Page 3
Figure S3	Page 4
Figure S4	Page 5
Figure S5	Page 6
Figure S6	Page 7
Figure S7	Page 8
Figure S8	Page 9
Table S1	Page 10
Table S2	Page 11
Table S3	Page 12
Table S4	Page 14
Table S5	Page 15


Figure S1. Interaction networks between plantivorous bats (red circles) and the plant taxa (green squares) present in their diet in the dry forest of Sector Santa Rosa (of Área de Conservación Guanacaste) (Costa Rica) and in the rainforest of La Selva Biological Station (Costa Rica), during an extreme El Ninõ year (2015). Networks for the whole-year and separated in the wet and dry seasons. Plant taxa were identified to genera or higher taxonomic levels.
1, Annona; 2, Bauhinia; 3, Bernardia; 4, Bromeliaceae; 5, Casearia; 6, Cecropia; 7, Columnea; 8, Epipremmum; 9, Erythroxylum; 10, Ficus; 11, Helicteres; 12, Juglandaceae; 13, Karwinskia; 14, Maclura; 15, Manilkara; 16, Moraceae; 17, Muntingia; 18, Ochroma; 19, Philodendron; 20, Pinus; 21, Piper; 22, Pourouma; 23, Sapotaceae; 24, Saxifragaceae; 25, Senna; 26, Solanum; 27, Vismia; 28, Zingiberales; 29, Artibeus jamaicensis; 30, Artibeus lituratus; 31, Dermanura tolteca; 32, Carollia castanea; 33, Carollia perspicillata; 34, Centurio senex; 35, Carollia sowelli; 36, Carollia subrufa; 37, Chiroderma villosum; 38, Dermanura phaeotis; 39, Dermanura watsoni, 40, Ectophylla alba; 41, Glossophaga sp.; 42, Lonchophylla robusta; 43, Micronycteris microtis; 44, Phyllostomus discolor; 45, Platyrrhinus helleri; 46, Uroderma convexum; 47, Vampyriscus nymphaea; 48, Vampyressa thyone.


Figure S2. Individual-based rarefaction curves comparing the species richness of plants present in the diet of plantivorous bat species during the dry season of an extreme El Niño year in the dry forest of Sector Santa Rosa (of Área de Conservación Guanacaste) (2015). Red line indicates the richness extrapolating 3 times the number of faecal samples analyzed for each bat species. 


Figure S3. Individual-based rarefaction curves comparing the species richness of plants present in the diet of plantivorous bat species during the wet season of an extreme El Niño year in the dry forest of Sector Santa Rosa (of Área de Conservación Guanacaste) (2015). Red line indicates the richness extrapolating 3 times the number of faecal samples analyzed for each bat species. 


Figure S4. Individual-based rarefaction curves comparing the species richness of plants present in the diet of plantivorous bat species during the dry season of an extreme El Niño year in the rainforest of La Selva Biological Station (2015). Red line indicates the richness extrapolating 3 times the number of faecal samples analyzed for each bat species. 


Figure S5. Individual-based rarefaction curves comparing the species richness of plants present in the diet of plantivorous bat species during the wet season of an extreme El Niño year in the rainforest of La Selva Biological Station (2015). Red line indicates the richness extrapolating 3 times the number of faecal samples analyzed for each bat species. 
Figure S6. Individual-based rarefaction curves comparing the species richness of plants present in the diet of plantivorous bat species during the whole-year of an extreme El Niño year in the rainforest of La Selva Biological Station (2015). Red line indicates the richness extrapolating 3 times the number of faecal samples analyzed for each bat species. 

Figure S7. Individual-based rarefaction curves comparing the species richness of plants present in the diet of plantivorous bat species during the whole-year of an extreme El Niño year in the dry forest of Sector Santa Rosa (of Área de Conservación Guanacaste) (2015). Red line indicates the richness extrapolating 3 times the number of faecal samples analyzed for each bat species. 


Figure S8. Individual-based rarefaction curves comparing the species richness of plants present in the diet of plantivorous bat species during the wet season of a non-El Niño year in the dry forest of Sector Santa Rosa (of Área de Conservación Guanacaste) (2009). Red line indicates the richness extrapolating 3 times the number of faecal samples analyzed for each bat species.


Table S1. Values of β dissimilarities between bat-plant networks during the dry and wet season in the dry forest of Sector Santa Rosa (Área de Conservación Guanacaste) and rainforest of La Selva Biological Station during an extreme El Niño event (2015) and a dry forest wet season of a non-El Niño year (2009). Plant taxa were identified at the lowest taxonomic level possible.
Differences	Dry forest – Rainforest	Dry forest Wet – Dry	Dry forest  El Niño wet season – non-El Niño wet season	Rainforest Wet – Dry
Dissimilarity in the species composition of the communities in the networks (βS)	0.58	0.42	0.53	0.50
				
Dissimilarity of interactions (βWN)	0.85	0.82	0.94	0.72
Dissimilarity of interaction established between species common to both networks (βOS)	0.50	0.58	0.78	0.46
Dissimilarity of interactions based due to species turnover between both networks (βST)	0.35	0.2438	0.16	0.26


Table S2. Sampling completeness of bat-plant networks during the dry and wet season in the dry forest of Sector Santa Rosa (Área de Conservación Guanacaste) and rainforest of La Selva Biological Station during an extreme El Niño event (2015) and a dry forest wet season of a non-El Niño year (2009). Plant taxa were identified at the lowest taxonomic level possible.
Type of forest (year – season)	Sampling completeness (%)
Dry forest (El Niño – whole-year)	86.44
Dry forest (Non-El Niño year – wet season)	78.85
Dry forest (El Niño – dry  season)	86.49
Dry forest (El Niño – wet season)	94.74
Rainforest (El Niño – whole-year)	79.71
Rainforest (El Niño – dry season)	95.65
Rainforest (El Niño – wet season)	83.17


Table S3. Mean, standard deviation and z-scores for the comparisons of bat-plant networks in the wet and dry season of the dry forest of Sector Santa Rosa (of Área de Conservación Guanacaste) and rainforest of La Selva Biological Station in Costa Rica during extreme an El Niño year (2015) with null models generated based on these networks. Additionally, for comparison, we present values for a network in the dry forest during the wet season of a non-El Niño year (2009). Plant taxa were identified at the lowest taxonomic level possible.
________________________________________________________________________________________________________________
1212  12 12	_________________Observed Values_________________	_________Vaznull - Null Model________	___Restricted Null Model___
Habitat - season	Modularity	WNODF	Connectance	Compartments	Plant species	Bat species	Total species	WNODF_SM	Modularity (mean)	Modularity (sd)	Modularity (z-score)	WNODF (mean)	WNODF (sd)	WNODF (z)	WNODF SM (mean)	WNODF SM (sd)	WNODF SM (z-score)
Dry Forest - Dry Season	0.57	0.07	0.18	3	16	11	27	0.22	0.43	0.05	2.77	0.16	0.04	-2.39	0.31	0.07	-1.43
Dry Forest - Wet Season	0.51	0.17	0.21	1	12	9	21	0.55	0.39	0.06	2.13	0.21	0.06	-0.71	0.45	0.10	0.28
Rainforest - Dry Season	0.55	0.07	0.20	2	16	7	23	0.34	0.51	0.06	0.70	0.12	0.04	-1.39	0.32	0.10	0.23
Rainforest - Wet Season	0.47	0.15	0.16	2	20	13	33	0.37	0.39	0.04	2.25	0.20	0.04	-1.12	0.26	0.06	2.03
Dry Forest - Whole-year	0.53	0.14	0.19	2	22	12	34	0.33	0.34	0.03	5.90	0.24	0.04	-2.13	0.41	0.06	-1.95
Rain Forest - Whole-year	0.46	0.14	0.15	2	29	13	42	0.32	0.35	0.03	3.72	0.20	0.03	-2.11	0.34	0.05	0.31
Dry Forest - Wet Season (Non- El Niño Year)	0.51	0.15	0.20	1	20	10	30	0.32	0.29	0.04	6.36	0.24	0.04	-2.30	0.32	0.05	-0.10


______________________________________________________________________________________________________________


1313
Table S4. Network metrics of bat-plant networks in the wet and dry season of the dry forest of Sector Santa Rosa (of Área de Conservación Guanacaste) and rainforest of La Selva Biological Station in Costa Rica and extreme an El Niño year (2015). Additionally, for comparison, we present values for a network in the dry forest during the wet season of a non-El Niño year (2009). Plant taxa were identified to genera or higher taxonomic levels.
 
Network metric	_____________Dry forest___________________ Whole-year     Dry               Wet        Wet non-                                                                    Niño year	__________ Rainforest________                                           Whole-year       Dry               Wet	
Plant richness	17	12	10	11	19	10	15	
Bat richness	12	11	9	10	13	7	13	
Number of compartments	2	2	1	1	2	2	2	
Weighted NODF	0.22	0.09	0.16	0.30	0.17	0.09	0.17	
Modularity	0.46	0.56	0.49	0.42	0.43	0.48	0.45	
Weighted NODFSM	0.45	0.28	0.47	0.36	0.33	0.08	0.41	
Connectance	0.22	0.22	0.26	0.27	0.17	0.21	0.17	

 


1414


Table S5. Values of dissimilarities between bat-plant networks during the dry and wet season in the dry forest of Sector Santa Rosa (Área de Conservación Guanacaste) and rainforest of La Selva Biological Station during an extreme El Niño event (2015) and a dry forest wet season of a non-El Niño year (2009). Plant taxa were identified to genera or higher taxonomic levels.

1515  15 15 Differences	Dry forest – Rainforest	Dry forest Wet – Dry	Dry forest El Niño wet season – non-El Niño  wet season	Rainforest Wet – Dry
Dissimilarity in the species composition of the communities in the networks (βS)	0.574	0.381	0.350	0.422
Dissimilarity of interaction established between species common to both networks (βOS)	0.448	0.429	0.394	0.500
Dissimilarity of interactions (βWN)	0.814	0.692	0.623	0.708
Dissimilarity of interactions based due to species turnover between both networks (βST)	0.366	0.264	0.229	0.208

 
